# Dynamics of Single-Chain Nanoparticles under Crowding: A Neutron Spin Echo Study

**DOI:** 10.1021/acs.macromol.4c00182

**Published:** 2024-05-07

**Authors:** Beatriz Robles-Hernández, Paula Malo de Molina, Isabel Asenjo-Sanz, Marina Gonzalez-Burgos, Stefano Pasini, José A. Pomposo, Arantxa Arbe, Juan Colmenero

**Affiliations:** † Donostia International Physics Center (DIPC), 20018 Donostia-San Sebastián, Spain; ‡ Centro de Física de Materiales/Materials Physics Center (CFM/MPC), 20018 Donostia-San Sebastián, Spain; § IKERBASQUE − Basque Foundation for Science, 48009 Bilbao, Spain; ∥ Forschungszentrum Jülich GmbH, Jülich Centre for Neutron Science (JCNS) at Heinz Maier-Leibnitz Zentrum (MLZ), 85748 Garching, Germany; ⊥ Department of Polymers and Advanced Materials: Physics, Chemistry and Technology, University of the Basque Country UPV/EHU, 20018 Donostia-San Sebastián, Spain

## Abstract

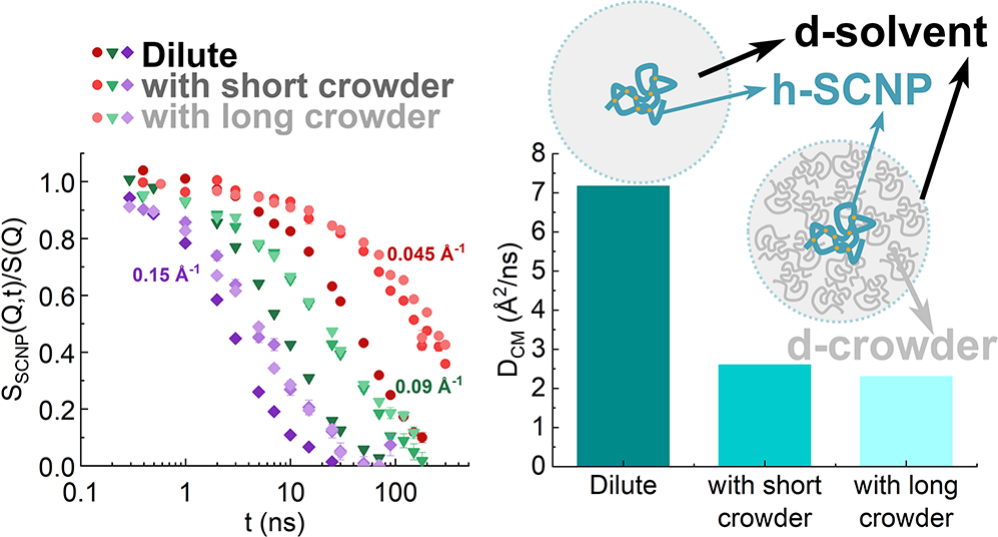

We present a neutron spin echo (NSE) investigation to examine the impact of macromolecular crowding on the dynamics of single-chain nanoparticles (SCNPs), serving as synthetic models for biomacromolecules with flexibility and internal degrees of freedom, such as intrinsically disordered proteins (IDPs). In particular, we studied the dynamics of a medium-size poly(methyl methacrylate) (PMMA)-based SCNP (33 kDa) in solutions with low- (10 kDa) and high- (100 kDa) molecular weight analogous deuterated PMMA linear crowders. The dynamic structure factors of the SCNPs in dilute solution show certain degrees of freedom, yet the analysis in terms of the Zimm model reveals high internal friction that effectively stiffens the chain—a phenomenon also observed for IDPs. Under crowding conditions, the internal dynamics remains essentially unchanged, but the center-of-mass diffusion slows down. The effective viscosity felt by the SCNPs at the timescales probed by NSE is lower than the macroscopic viscosity of the crowder solution, and it does not depend significantly on the molecular weight.

## Introduction

The high concentration present in the cellular environment, known as crowding, affects the biological function of biomacromolecules through changes not only in their structure but also in their dynamics.^1^ Not unexpectedly, macromolecular crowding leads to a slowing down of translational diffusion. In the case of protein solutions, the self-diffusion coefficient decreases with concentration at a rate that depends on the protein shape as well as on the intermolecular interactions.^2,3^ Moreover, the internal dynamics can also be affected by crowding at high concentrations.^4^ In fact, the effect of crowding conditions on the chain internal dynamics is of particular interest in the case of unfolded protein chains, including intrinsically disordered proteins (IDPs). Such proteins, owing to their inherent flexibility, are able to explore an extensive conformational landscape associated with protein folding and target binding.^5^

However, proteins exhibit an extensive range of compositions, leading to diverse molecular interactions. Consequently, the use of simplified synthetic models becomes imperative to discern the topological effects on dynamics from those arising from specific interactions. In this context, single-chain nanoparticles (SCNPs) prove to be ideal model systems for IDPs in both dilute and concentrated solutions.^6^ Essentially, SCNPs represent polymer chains that collapse through intramolecular bonding between reactive functional sites, forming internal loops and reducing the chain’s overall size. The cross-linking reaction is conducted under high dilution to prevent undesired intermolecular bonding.^7^ In good solvent conditions, the formation of short-range loops is favored over long-range loops due to the self-avoiding conformation of the chain, resulting in sparse, nonglobular SCNPs in solution.^8^ Moreover, unlike globular proteins, whose folding is driven by defined interactions, the collapse of synthetic SCNPs occurs through a stochastic process. Consequently, compaction is less controlled, leading to a polydispersity of resulting topologies.^8,9^

SCNPs have served as model systems for investigating the impact of macromolecular crowding on structure through small-angle neutron scattering (SANS). Specifically, an examination of the influence of crowding induced by linear polystyrene (PS) of both low- and high-molecular weights on the structure of PS-based SCNPs revealed different behaviors based on the crowder molecular weight. Long crowder chains led to SCNP compression above their overlap concentration, while short ones were found to create depletion interactions leading to aggregation.^10^ In a separate study, the effect of poly(methyl methacrylate) (PMMA) on the structure of PMMA-based SCNPs was investigated using SANS in combination with molecular dynamics (MD) simulations. The SCNP conformation exhibited a transition from unperturbed dimensions in dilute conditions to a more collapsed state when the total polymer concentration surpassed the SCNP overlap concentration, decreasing further in size with increasing crowder concentration, being this collapse more apparent for higher-*M*_w_ SCNPs.^11^ A comprehensive SANS analysis using a random phase approximation for similar systems revealed that the Flory–Huggins interaction parameter in the dilute regime suggests that the precursors and the SCNPs are in good solvent conditions, whereas in crowding conditions, the polymer becomes less soluble.^12^ Now, the objective is to elucidate the effect of crowding on the dynamics of the SCNPs in those same systems.

Probing the dynamics in the concentrated regime is challenging. Techniques often used to investigate the dynamics of NPs in polymer solutions, such as dynamic light scattering (DLS) and sedimentation, are unsuitable for polymer mixtures in solution due to the similarities in density and dynamic timescales of probe and crowder. Instead, the study of the dynamics of small amounts of polymeric molecules or NPs in crowded environments requires the use of experimental techniques with labeling; for instance, fluorescence correlation spectroscopy (FCS),^13^ fluorescence recovery after photobleaching (FRAP),^14^ or nuclear magnetic resonance (NMR).^15^ However, these techniques mostly probe longer timescales but, more importantly, they lack spatial resolution. In contrast, neutron scattering (NS) techniques have both temporal and spatial resolution, which allows to identify the nature of the dynamic process as well as quantify its timescale. Furthermore, isotopic labeling with hydrogen and deuterium enables the enhancement of the contribution of small quantities of one species in the presence of a high concentration of other molecules. For this reason, NS is ideal for studying the internal and diffusive dynamics of biomolecules in dilute and concentrated solutions.^16^ Specifically, neutron spin echo (NSE) is particularly well suited to probe the dynamics of flexible chains in solution due to the timescale window—given by the excellent energy resolution of the technique—along with the accessible length scales—given by the magnitude of the scattering *Q*-vector. Thus, it allows exploring the polymer chain dynamics, which has, in general, a hierarchical nature ranging from backbone and side chain fluctuations to the center-of-mass diffusion.^17,18^

Previous NSE studies on the dynamics of SCNPs in dilute solutions revealed the relaxation of internal degrees of freedom, but clearly slowed down with respect to their linear precursor counterparts.^19,20^ This effect is attributed to the internal friction associated with the compartmentalization in domains within the macromolecule. To describe the dynamic structure factor, the dual polymer/NP character of the SCNPs in solution was considered by applying theoretical approximations based on the Zimm model.^19,20^ Similar dynamic behavior has been found from NSE investigations on solutions of IDPs^21^ and protein chains unfolded by denaturing,^22−24^ with a relatively large contribution of internal dynamics to the overall diffusion but a slowing down of the more local modes. This effect can be taken into account by adding an internal friction and is essentially the same effect found in SCNPs. Thus, SCNPs can be considered good models to study now the effect of crowding on the dynamics of biologically relevant macromolecules.

In this work, we explore the dynamics of our model SCNPs in crowded solutions with analogous linear polymers using NSE combined with isotopic labeling. In this way, we access the single-chain dynamics of H-labeled NPs in a solution of deuterated linear polymers and deuterated solvent. Specifically, we use PMMA-based SCNPs of 33 kDa (the mass of an intermediate-size protein), and dPMMA crowders with two molecular weights: 10 and 100 kDa. After dwelling on the structure, we present a phenomenological analysis of the NSE data, followed by an analysis of the intermediate scattering functions in terms of the Zimm model for polymer chains.

## Materials and Methods

### Single-Chain Nanoparticle Synthesis and Sample Preparation

**Scheme 1:**
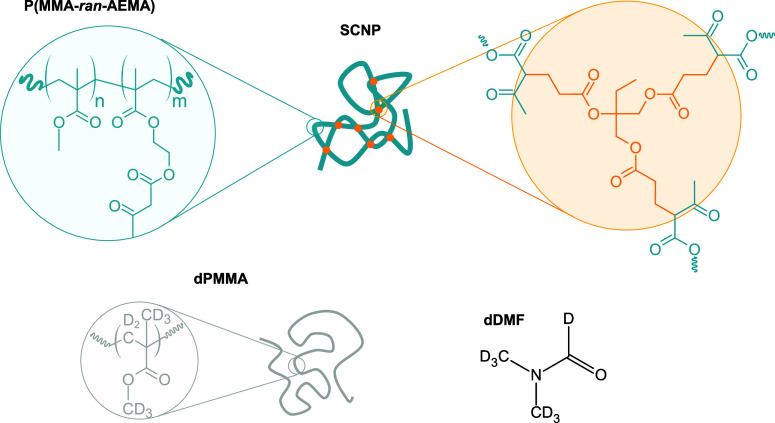
Chemical Structure of the Poly(methyl methacrylate-*ran*-(2-acetoacetoxy)ethyl methacrylate) SCNPs, the dPMMA Crowder Chains and the dDMF Solvent In the samples studied in this work, *n* = 0.69 and *m* = 0.31.

The linear precursor for SCNP preparation consisted of a random copolymer of methyl methacrylate (MMA) and (2-acetoacetoxy)ethyl methacrylate (AEMA), specifically P(MMA_0.69_-*ran*-AEMA_0.31_), synthesized via reversible addition–fragmentation chain-transfer polymerization in a process described previously.^25^ The SCNPs were obtained through Michael addition of the trifunctional cross-linking agent trimethylolpropane triacrylate (TMT, 33 mol % to AEMA) (Sigma-Aldrich, technical grade) to the β-ketoester functional groups of the precursors^25^ (see Scheme 1). The molecular weight and dispersity of the sample as determined by size exclusion chromatography with multiangle laser light scattering (SEC/MALLS), together with other physicochemical parameters, are displayed in Table 1.

**Table 1:** Molecular Characteristics of SCNPs and Crowders: Molecular Weight (*M*_w_) and Dispersity (*D̵*), Radius of Gyration (*R*_g_), Scaling Exponent (ν), Overlap Concentration (c*), and Scattering Length Density (ρ)

	*M*_w_^a^ (kg/mol)	*D̵*^a^	*R*_g_^b^ (nm)	ν^b^	c*^c^ (mg/mL)	ρ (10^10^ cm^–2^)
SCNPs	33.9	1.04	4.3	0.39	90	1.27
crowded with Lo-dPMMA			4.4	0.40		
crowded with Hi-dPMMA			4.3	0.38		
Lo-dPMMA	9.6	1.1	3.4	0.59	51	6.97
Hi-dPMMA	99.1	1.09	10.9	0.59	16	6.97

a From SEC/MALLS in THF.

b From SANS.^12^

c .

The solvent for neutron scattering experiments was deuterated *N*,*N*-dimethylformamide (dDMF, 99.5 atom %, Acros Organics). Crowded solutions were prepared by adding deuterated linear PMMA chains of two different molecular weights (dPMMA, Polymer Source, see Table 1). After synthesis and purification, stock solutions of SCNPs were prepared, and the required amount of dPMMA was added immediately to reach the desired total concentration, which for SCNPs crowded solutions is *c*_tot_ = *c*_SCNP_ + *c*_crowder_ = 20 mg/mL + 180 mg/mL = 200 mg/mL. As a reference, a SCNP solution at 20 mg/mL without crowders (we will refer to it as dilute solution) was investigated. The concentration of the SCNPs is always below the overlap concentration, estimated as , while in the crowded samples, the total concentration of polymer is above the overlap concentration of the SCNPs. For viscosity measurements, solutions of Hi-dPMMA (see Table 1) and Lo-hPMMA (*M*_w_ = 11.5 kg/mol, Polymer Source) in DMF at different concentrations were prepared.

### Neutron Spin Echo

NSE experiments were carried out using the J-NSE instrument at the MLZ.^26^ Combining three wavelengths λ (8, 10, and 12.5 Å), Fourier times in the range 0.1 ≤ *t* ≤ 300 ns were covered in the *Q*-range (*Q* = 4π sin θ/λ, where 2θ is the scattering angle) 0.03 ≤ *Q* ≤ 0.15 Å^–1^. Solutions of SCNPs in dDMF in the dilute regime as well as in crowded conditions with deuterated linear PMMA were measured at a temperature of 300 K. We consider the background signal from any coherent and incoherent non-SCNP scattering, i.e., it has contributions from the solvent molecules and the crowder polymer chains. Thus, the NSE signal for the SCNPs is obtained after subtracting the corresponding background signal measured on the crowder solutions prepared at the same concentrations and measured with equal statistics as that of the sample.

NSE measures the loss of polarization due to the dephasing of the neutron spins in time, allowing energy resolutions of the order of neV, thereby reaching Fourier times of up to many 100 ns. However, the incoherent scattering has a 2/3 probability to spin-flip the scattered neutrons converting them into a “non-polarized” background. Therefore, the total NSE signal—the normalized intermediate scattering function *S*(*Q*, *t*)/*S*(*Q*, 0)—is given by *S*_NSE_(*Q*, *t*) = [*I*_coh_*S*_coh_(*Q*, *t*) – 1/3*I*_inc_*S*_inc_(*Q*, *t*)]/[*I*_coh_ – 1/3*I*_inc_], where *S*_coh_(*Q*, *t*) and *S*_inc_(*Q*, *t*) are intermediate pair correlation functions, normalized to their value at *t* = 0, and *I*_coh_ and *I*_inc_ are the total static coherent and incoherent intensities detected by the instrument.

### Dynamic Light Scattering

The diffusion coefficients of the SCNPs in dilute conditions were determined from DLS experiments. The scattering vector *Q* is given by *Q* = 4π*n*_d_ sin θ/λ_0_, where λ_0_ is the wavelength in vacuum and *n*_d_ is the solvent refractive index. The experiments were carried out on a Malvern Zetasizer Nano ZS apparatus at 300 K. Solutions of SCNPs in DMF at the same concentration as the NSE samples were investigated. The *Q*-value explored with the experimental setup (2θ = 173°, λ_0_ = 633 nm, *n*_d_(DMF) = 1.431) was 0.00284 Å^–1^.

### Small Angle X-ray Scattering

Small-angle X-ray scattering (SAXS) experiments in solutions of deuterated PMMA crowders in deuterated DMF were carried out at room temperature on a Rigaku 3-pinhole PSAXS-L instrument operating at 45 kV and 0.88 mA. The MicroMax-002+ X-ray generator system is composed of a microfocus sealed tube source module and an integrated X-ray generator unit that produces Cu Kα transition photons of wavelength λ = 1.54 Å. The flight path and the sample chamber were under vacuum. The scattered X-rays were detected on a two-dimensional multiwire X-ray detector (Gabriel design, 2D-2000×). This gas-filled proportional type detector offers a 200 mm diameter active area with ca. 200 μm resolution. The azimuthally averaged scattered intensities were obtained as a function of the scattering vector. Reciprocal space calibration was done using silver behenate as the standard. The solutions were filled in boron-rich capillaries with an outside diameter of 2 mm and a wall thickness of about 0.01 mm. The sample was placed in transmission geometry, with a sample to detector distance of 2 m, covering a *Q*-range between about 0.01 and 0.2 Å^–1^. Each sample was measured for 1 h. The solvent was measured under the same conditions and properly subtracted from the measurements on the solution.

### Viscosity Measurements

The viscosities of solutions of crowders in DMF at different concentrations (in the range from 10 to 200 mg/mL) were measured with an electromagnetically spinning viscometer EMS-1000 (Kyoto Electronics, Kyoto, Japan) operating at 300 K. The viscosity of dDMF was measured using a calibrated micro-Oswald capillary viscometer (Schott) type I at 300 K.

## Results and Discussion

### Prior Structural Considerations

In the NSE experiments, the scattering of the hydrogenated SCNPs is enhanced due to the low contrast between the deuterated crowders and the deuterated solvent. Indeed, the neutron scattering length density ρ of dDMF is 6.36 × 10^10^ cm^–2^, close to that of dPMMA (see Table 1). Before dwelling on the dynamics, let us consider the structure of the system in the relevant length scales. The structure of unordered systems like those here investigated is best probed with SANS. SCNPs in solutions generally exhibit a polymer coil-type structure, which is more compact and smaller than their linear precursor counterparts and can be modeled with a generalized Gaussian coil form factor.^19^ In crowded solutions with homologous linear chains, several studies have shown that high-molecular weight SCNPs compress when the total polymer concentration is higher than the overlap concentration of the SCNPs,^10,11^ and the best analysis takes into account the polymer–solvent interactions.^12^

In a recently published structural study on the samples here investigated,^12^ a random phase approximation (RPA) approach was used to describe the SANS intensity. That analysis takes into account the three components in the system (SCNPs, crowder, and solvent) and the polymer–solvent interactions between PMMA and DMF. Then, the scattered intensity is given as the summation of squares of scattering length density differences Δρ_*i*_ between polymer chains and solvent molecules, multiplied by the fully interacting system structure factors
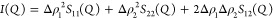
1

The fully interacting system structure factors *S*_*ii*_(*Q*) depend on the single-chain form factors *S*_*ii*_^0^(*Q*) = *N*_*i*_ϕ_*i*_*v*_*i*_*P*_*i*_(*Q*) (with *N*_*i*_ being the degree of polymerization of the component *i*, ϕ_*i*_ its volume fraction, *v*_*i*_ its molar volume, and *P*_*i*_(*Q*) its form factor) and the interaction parameter χ between the polymer and the solvent (see ref (12)).

**Figure 1:**
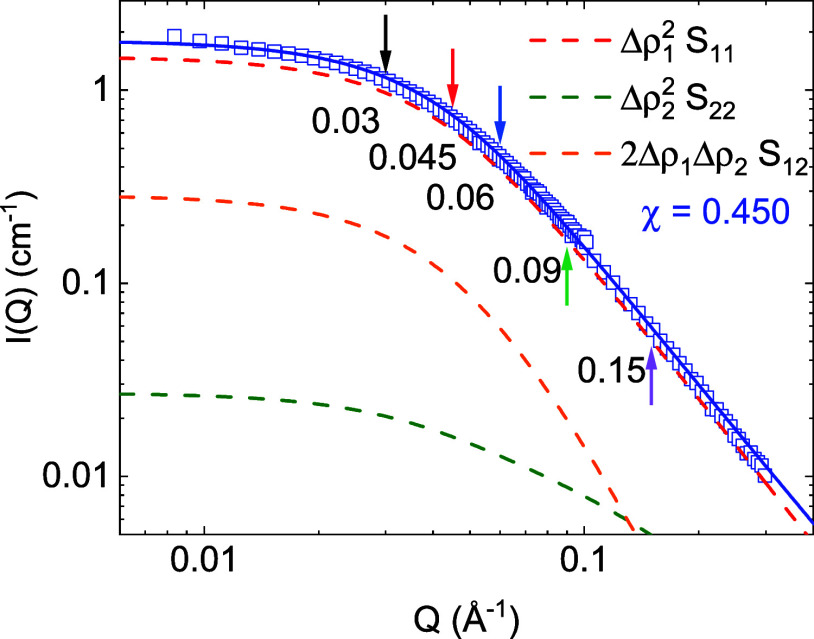
Small-angle neutron scattering (SANS) intensities *I*(*Q*) of SCNPs in crowding with Lo-dPMMA (*c*_tot_ = *c*_SCNP_ + *c*_crowder_ = 20 mg/mL + 180 mg/mL = 200 mg/mL) with RPA fits (solid line). Each contribution on eq 1 is represented in a different color (dashed lines, see legend). Arrows mark the *Q*-values (in Å^–1^) at which the NSE experiment was carried out. Adapted with permission from ref (12) Copyright 2023, American Chemical Society.

The results of such an analysis show that the contributions of the crowder and the cross-term to the scattered intensity are non-negligible at low *Q*. However, in the *Q*-range explored in the NSE measurements, the contribution to the scattered intensity comes mainly from the SCNPs, with a negligible amount of signal coming from the crowder and the cross-term (see Figure 1 for the SCNPs crowded with Lo-dPMMA). In addition, the NSE signal is obtained after subtracting the background signal measured on crowder solutions at the same concentration, and the NSE contribution from incoherent scattering in these systems is also negligible. Thus, we can safely assume that the single-chain dynamic structure factor obtained in the NSE experiments corresponds to the SCNP dynamics
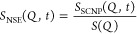
2The denominator *S*(*Q*) is the *t* ≃ 0 limit of the single-chain dynamic structure factor *S*_SCNP_(*Q*, *t*) (see, e.g., refs (18, 27, 28)).

For this system, the radius of gyration of the SCNPs is about 4.3 nm both in dilute and crowding conditions, and the scaling exponent remains close to ν ∼ 0.4 for all solutions (see Table 1). In the present case, the SCNP does not change its size significantly upon crowding. This is consistent with the low-molecular weight and the very collapsed conformation of the SCNP already in dilute solutions. We note that in the systems crowded with PMMA chains, the total polymer concentration is higher than but close to the SCNP overlap concentration (see Table 1), and the relative size reduction of the SCNPs upon crowding is minimal. On the other hand, the Flory–Huggins interaction parameter varies with composition: in the dilute regime, DMF is a good solvent for PMMA, while in crowded conditions, the polymer–solvent interactions become less favorable.^12^

### Dynamics

**Figure 2:**
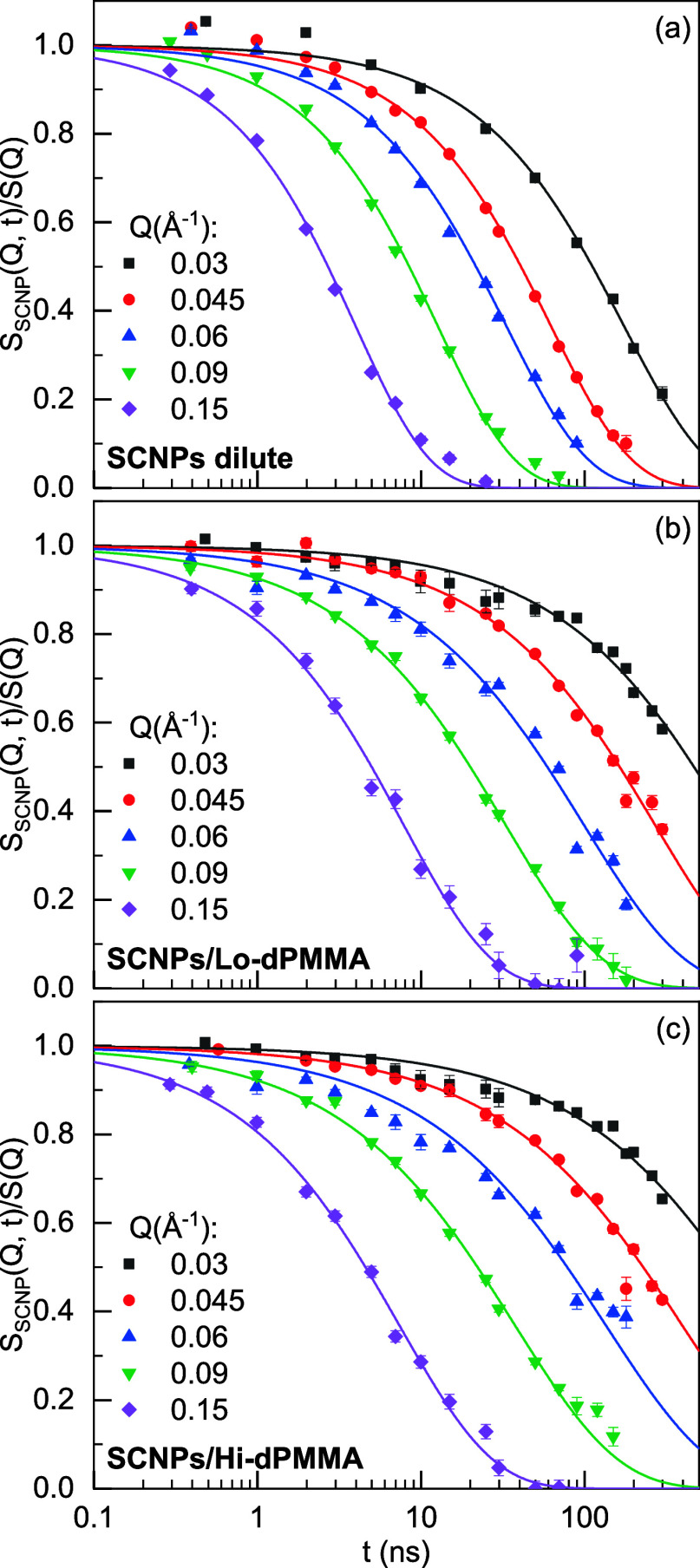
NSE measured intermediate scattering function of SCNP solutions (a) in the dilute regime, and under crowding conditions with (b) Lo-dPMMA and (c) Hi-dPMMA, at the different *Q*-values indicated. Lines are fits to eq 3.

Figure 2 displays the NSE results of the SCNP solutions in dilute and crowded conditions with low- and high-molecular weight dPMMA crowders. The first observation of the data reveals that the decay of the dynamic structure factor of the crowded samples is slower than that of the dilute sample for all the *Q*-values probed by NSE. We initially provide a preliminary inspection of the impact of crowding on the dynamics, which should give a notion of the origin of the contributions at the different length scales investigated. To do so, we analyzed the obtained results following a simple phenomenological approach to describe the dynamics.

#### Phenomenological Approach

**Figure 3:**
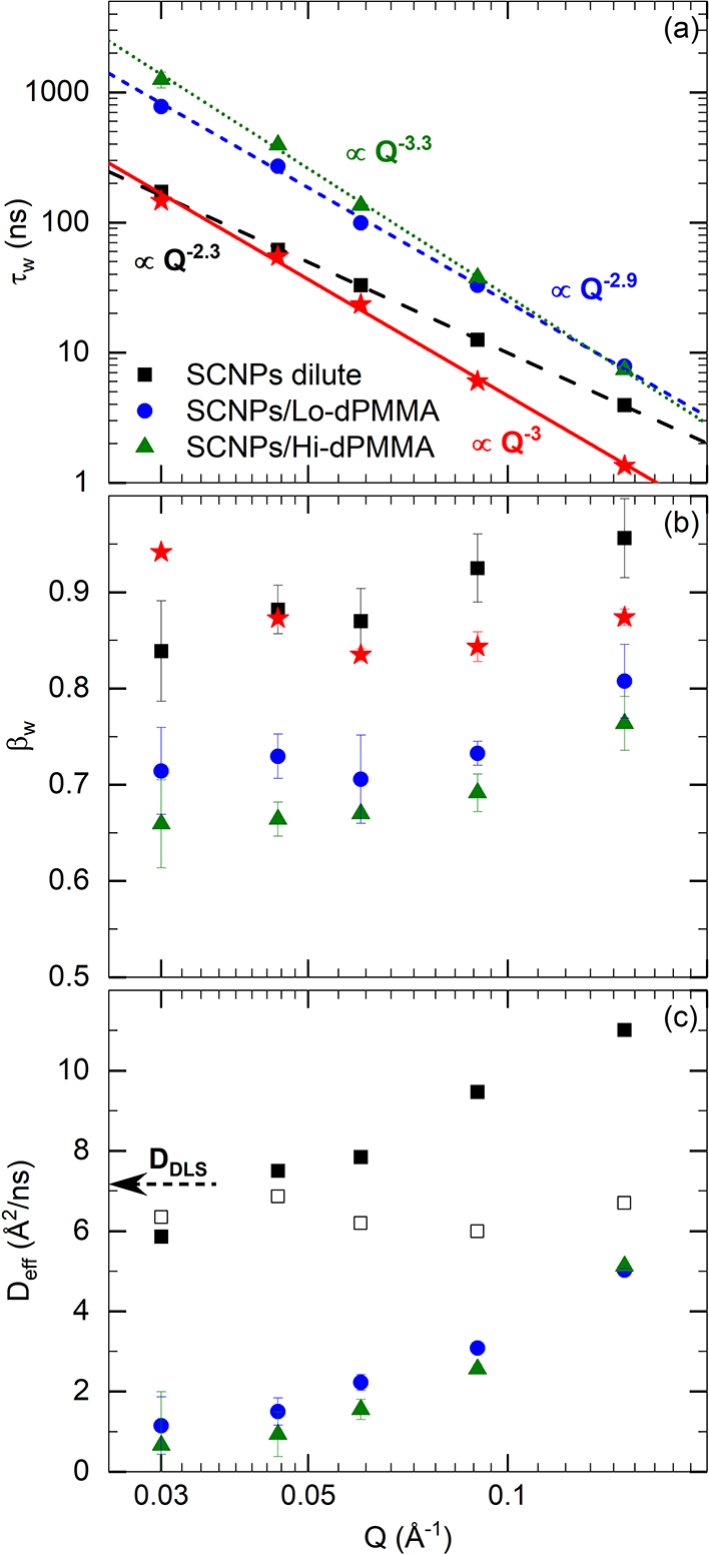
Dynamic parameters obtained with eq 3 as a function of the scattering vector for SCNPs in dilute conditions (black squares) and in crowded conditions with Lo-dPMMA (blue circles), and with Hi-dPMMA (green triangles). Red stars correspond to a stretched exponential description of the pure Zimm model results with the same diffusion coefficient as in the dilute case. (a) Characteristic time; (b) stretching exponent; and (c) effective diffusion coefficient. Open symbols are the center-of-mass diffusion coefficients obtained from Zimm model-based analysis (see text). Lines in (a) are fits to power laws with exponents as indicated. Arrow in (c) marks the value of *D*_DLS_ for the SCNPs in dilute solution.

As a first approximation, the apparent diffusivities are obtained from an analysis in terms of stretched exponential functions
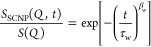
3where τ_w_ is the relaxation time and β_w_ is the stretching exponent. In the case of simple diffusion, the single exponential expression is recovered (β_w_ = 1), and the diffusion coefficient *D* can be obtained as *D* = τ_w_^–1^*Q*^–2^. The values of β_w_ < 1 indicate a distribution of relaxation processes or deviations from simple diffusive motions. Figure 2 shows that using eq 3 a good description of the data is obtained. The values of τ_w_ and β_w_ obtained from these fits are represented as a function of *Q* in Figure 3a,b. As can be observed, the *Q*-dependence of τ_w_ deviates from those corresponding to simple diffusion. In addition, β_w_ deviates from unity as well. This is a signature of the presence of additional contributions superimposed to the SCNP translational diffusion, namely, internal degrees of freedom contributing to the dynamic structure factor at the length scales investigated by NSE.

**Figure 4:**
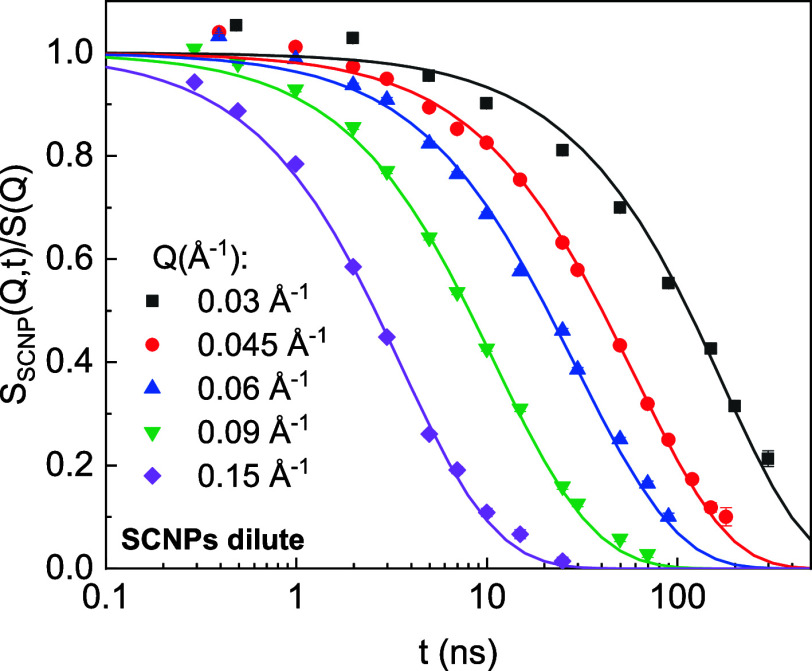
NSE results for SCNPs in dilute solution. Solid lines are fits considering a Zimm model with limited mode contributions (*p*_max_ = 1). See text for details on the assumptions and approaches.

Neutral flexible polymer chains generally exhibit a Zimm-type dynamics, characterized by relaxation times that scale as τ_w_ ∼ *Q*^–3^, with β ∼ 0.85 in the *R*_g_*Q* ≫ 1 range^17,37^ (see stars in Figure 3a,b). The obtained relaxation time on the dilute solution of SCNPs has a *Q*-dependence of *Q*^–2.3^, suggesting a slowing down of the internal dynamics at the local length scale, as previously observed for SCNPs.^19,20^ When crowding is introduced in the system, the β_w_ parameter reaches values markedly lower than Zimm’s prediction. Besides, the *Q*-dependence of the relaxation time increases. At low *Q*, the relaxation time has to be essentially dictated by diffusion, while at high *Q*, it reflects internal dynamics. Thus, the steeper *Q*-dependence would be related to the fact that the internal dynamics and the diffusion separate in time.

To compare the results from the SCNPs in dilute and in crowded solutions at different *Q*-values, we have defined an effective diffusion coefficient *D*_eff_(*Q*) = ⟨τ⟩^–1^*Q*^–2^, where the average time ⟨τ⟩ for each *Q*-value is expressed in terms of the gamma function of the inverse of the stretching parameter as ⟨τ⟩ = τ_w_Γ(1/β_w_)/β_w_. The values of *D*_eff_ are plotted in Figure 3c and, as can be observed, *D*_eff_(*Q*) is smaller for the SCNPs in crowded solutions than for those in dilute solutions. Thus, there is a slowing down of the dynamics upon crowding with linear dPMMA chains. Interestingly, the reduction in diffusivity only depends moderately on the crowder molecular weight, being slightly more pronounced in the case of Hi-dPMMA. This is surprising since it suggests that the effective viscosity felt by the SCNPs is similar in both crowded solutions, despite the macroscopic viscosities of both crowder solutions differing in an order of magnitude (see below).

In Figure 3c, the arrow indicates the value of the diffusion coefficient determined on the dilute sample (at a much smaller scattering vector) from the DLS experiments (*D*_DLS_ = 7.17 Å^2^/ns). In such a low-*Q*-regime explored by DLS, the SCNPs can be considered as point particles subjected to Brownian motion, and the obtained diffusion coefficient corresponds to the center-of-mass translation. In fact, DLS delivers the collective diffusion coefficient, related to the self-diffusion coefficient at infinite dilution *D*_0_ through the structure factor *S*(*Q*) and the hydrodynamic function *H*(*Q*): *D*(*Q*) = *D*_0_*H*(*Q*)/*S*(*Q*).

In the dilute solution of SCNPs, the *D*_eff_(*Q*) values determined from NSE increase with *Q*, reaching values larger than *D*_DLS_. This is a signature of enhanced mobility, as mentioned before, due to the contributions of internal modes, which become apparent when exploring the proper length scales by NSE. The internal modes’ contribution progressively increases with the *Q*-value (as the length scale of observation becomes smaller). In the region *QR*_g_ ≫ 1, the Zimm model for neutral dilute polymer chain dynamics predicts a linear *Q*-dependence of the effective diffusion coefficients.^17^ Again, deviations from the Zimm dynamics reflect the internal friction-induced stiffness of the chain that produces a slowdown of the local modes.^29^ On the other hand, in the crowded samples, although *D*_eff_ is always lower than in dilute conditions, the trend for *D*_eff_ with increasing *Q*-values is analogous. Thus, this phenomenological approach ultimately indicates that the contribution of the chain internal dynamics to the overall diffusion remains upon crowding and that the presence of the crowder results mainly in a slowing down of the dynamics.

#### Analyses Based on the Zimm Model

SCNPs are macromolecules in-between colloidal objects and polymeric entities. Thus, their particle nature has an influence on their center-of-mass diffusion at length scales (*Q*-values) at which the intermolecular interactions start to be noticeable. Conversely, the softness and internal loop structure of SCNPs entail that chain strands belonging to flexible domains of the macromolecule relax and, therefore, contribute to the decay of the dynamic structure factor. Taking into account these considerations, NSE results from SCNPs in dilute conditions have been successfully described using the Zimm model,^30^ which describes the dynamics of flexible chains in dilute conditions,^17,18^ under certain assumptions.^20^ First, we consider that the translational diffusion characterized by the center-of-mass diffusion, *D*_CM_, and the internal motions take place simultaneously and independently. Under this assumption, the single-chain dynamic structure factor can be expressed as the product of both contributions
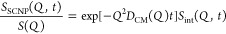
4with *S*_int_(*Q*,*t*) being the normalized dynamic structure factor corresponding to the internal motions of the macromolecule, which can be described using the Zimm model.

This model considers a coarse-grained chain composed of *N* beads connected by entropic springs of length *l*. The beads are affected by hydrodynamic interactions mediated by the solvent of viscosity η_0_. The resulting Langevin equation can be solved by transforming to the Rouse coordinates defined as
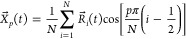
5Here,  is the position vector of the *i*-th bead along the chain and *p* is the mode number (*p* = 0, ..., *N* – 1). The zeroth *p*-mode corresponds to the center of mass of the chain, and the others are associated with internal motions with a wavelength *lN*/*p*. The mode correlators decay exponentially with characteristic times τ_*p*_^Z^ given by
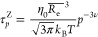
6where *k*_B_ is the Boltzmann constant and  is the end-to-end distance, which can be calculated as^31^
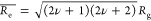
7with *R*_g_ being the radius of gyration and ν being the scaling exponent.^32,33^ Thus, the higher the mode number, the more localized it is, and the faster it decays. The Zimm dynamic structure factor is expressed as
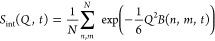
8in terms of the correlators *B*(*n*, *m*, *t*)
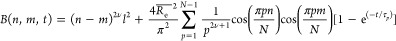
9

In real systems, the Zimm model only delivers a reasonable description for low-*Q* values, while on approaching local length scales, the dynamics deviates from the Zimm prediction. Indeed, the bare Zimm model seems to describe the dynamic structure factor properly up to high *Q*-values only for extremely flexible polymers,^34,35^ while for other polymers in solution (e.g., polyisobutylene^34^ and polynorbornenes^35^), it predicts a much more steep decay at higher *Q*-values, i.e., when approaching local length scales. In particular, this was also found in PMMA-based SCNPs.^19,20^ These deviations can be attributed, to a large extent, to dynamical stiffness,^34−37^ which can be introduced in the model by limiting the modes contributing to the chain relaxation (*p*_max_ as the upper limit in the sum of eq 9). This mode cutoff could be interpreted in terms of virtually rigid subcoils, with all internal modes suppressed.^35^ From the value of *p*_max_, the size of the virtual stiff chain can be estimated, with an average end-to-end radius .^35^

The dynamic structure factor obtained from NSE for the dilute solution of SCNPs was analyzed in terms of the Zimm model with mode cutoff using eq 4 with *D*_CM_(*Q*) as a fitting parameter. The SCNP has been mapped to an effective linear chain with the same scaling exponent and dimension as deduced from SANS^12^ (see Table 1). The validity of this approximation is supported by MD simulations of dilute SCNPs,^19^ where the normal modes of the effective chains were calculated, and the relaxation time of the *p*th mode, τ_*p*_, was obtained. It was found that, for long wavelengths, τ_*p*_ is consistent with the Zimm scaling (τ ∼ *p*^–3ν^), where ν is the exponent obtained from the fractal regime in the form factor. Assuming the length of the beads as the statistical segment deduced for this copolymer (*b* = 3.27 nm)^19^ and taking into account the value of  nm obtained from SANS results^12^ through eq 7, the equivalent chain would consist of *N* = 16 segments . For τ_*p*_^Z^ in 6 we consider the viscosity of dDMF at 300 K, η_0_ = 0.82 mPa s.

A good description of the experimental data was obtained with *p*_max_ = 1 (see Figure 4), in agreement with results obtained in the same system previously.^20^ Such a low value of *p*_max_ essentially means that the internal dynamics of the internally cross-linked macromolecule is extremely hindered, with only the rotational mode persisting. The virtually rigid subcoil is the whole SCNP. This may seem surprising but is quite in agreement with the low value found for the scaling exponent ν, close to the limit value of 0.3 for globules (see Table 1). The restriction of the Zimm modes contributing to the chain relaxation imposed by the mode cutoff can be seen as a consequence of the internal friction concept used in the Zimm model with internal friction (ZIF).^38^ Indeed, a very good description of the data is obtained using the ZIF model with an internal friction τ_i_ = 55 ns, in agreement with the previous investigations on similar SCNPs.^19,20^ In the Supporting Information, the outcome of this description can be found, together with a comparison of the characteristic times involved in each of the models.

The diffusion coefficients obtained from the fitting of eq 4 with a mode-cutoff Zimm model to the SCNP dilute solution data are represented in Figure 3c as open symbols. In a previous study on the dynamics of PMMA-based SCNPs on dilute solutions,^20^ the *Q*-dependence of the diffusion coefficients showed a minimum mirroring the broad maximum of the structure factor (see Figure 5 in ref (20)), attributed to the de Gennes narrowing and reflected by the slowing down of the collective diffusion at length scales corresponding to equilibrium interparticle distances. In the present case, the same trend for the diffusion coefficient is expected. The similar values obtained for the effective diffusion and the center-of-mass diffusion of the SCNPs in dilute conditions at *Q* = 0.03 Å^–1^ indicate that the dynamic structure factor of this system at such low-*Q* values is dominated by the translational diffusion component.

**Figure 5:**
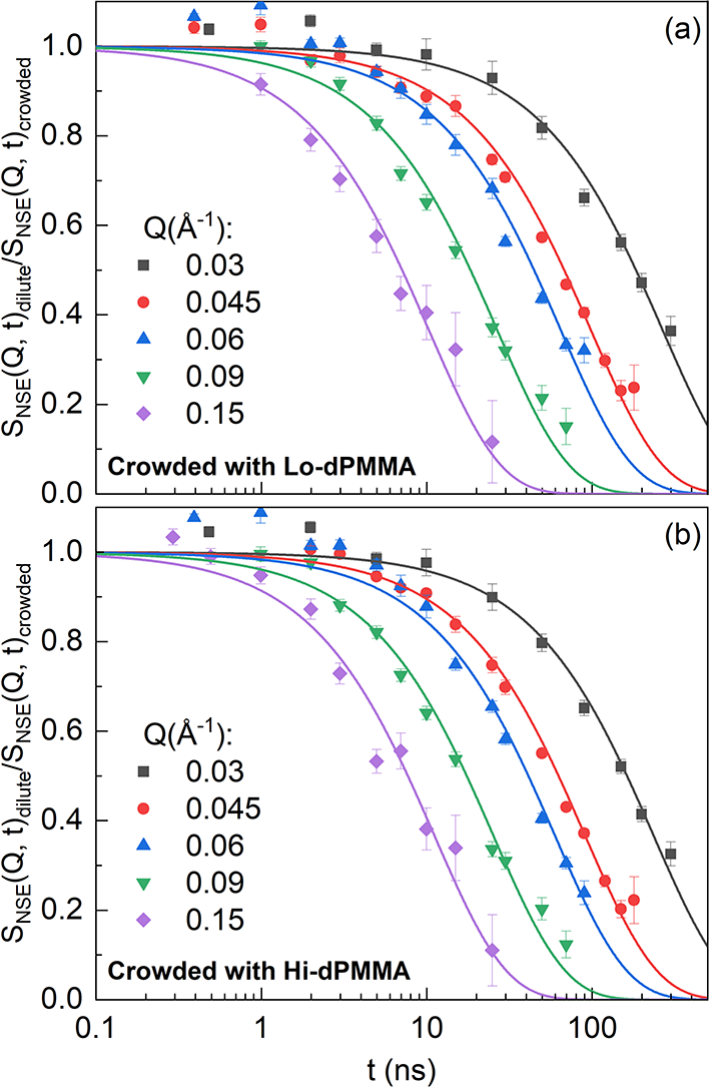
Quotient of the NSE results from SCNPs in dilute conditions divided by those in crowding conditions with (a) the low-*M*_w_ and (b) the high-*M*_w_ linear dPMMA. Lines are fits to an exponential decay function (see eq 10).

We now move on to the dynamics of SCNPs in the presence of crowders. A priori, the internal dynamics of the SCNPs is not expected to dramatically change under crowding conditions. The internal modes would be affected by the shrinking produced by the crowded environment (change in the modes, eqs 9 and 6, if the ν-value decreases), but the collapse of this system is minimal.^12^ In addition, in the SCNPs here investigated, even in dilute conditions, only the rotational mode persists—no further internal modes are left to be suppressed under crowding. Thereby, crowding would have its main impact on the dynamics related to the center-of-mass diffusion of the SCNP.

As mentioned before, these SCNPs in dilute conditions have already a very collapsed conformation, and structural investigations on these samples have revealed that the form factor parameters do not significantly change upon crowding^12^ (see Table 1). Thus, under these circumstances, it can be assumed that *S*_int_(*Q*,*t*)_dilute_ = *S*_int_(*Q*,*t*)_crowded_, and, according to eq 4, the quotient of experimental results on dilute conditions divided by those on crowded solutions can be expressed as
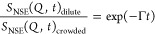
10where Γ = *Q*^2^(*D*_CM_^dilute^ – *D*_CM_^crowded^). In this way, upon the determination of the center-of-mass diffusion of SCNPs on the dilute solution, the slowing down of the translational diffusion due to the presence of linear crowders can be extracted. Equation 10 suggests that the quotient of experimental data should have the functional form of a monoexponential decay.

**Figure 6:**
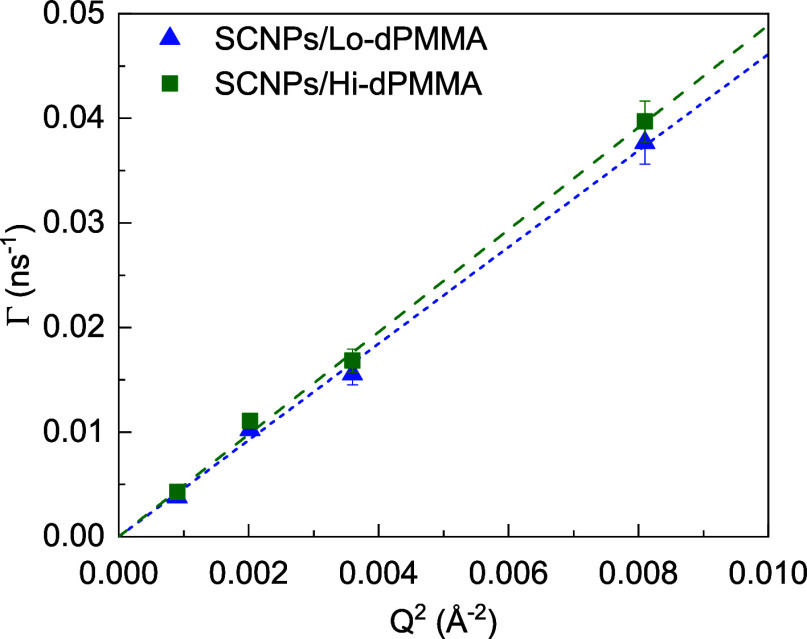
Decay constant Γ obtained from the data in Figure 5 for the SCNPs in crowded conditions with Lo-dPMMA (blue triangles) and Hi-dPMMA (green squares) linear crowders as a function of *Q*^2^. Dashed lines are linear fits (see text).

Figure 5 displays the curves obtained upon the division of the SCNP data in the dilute solution by the data obtained in crowded conditions. As can be seen, for the measured *Q*-range, all the curves can be described by a single exponential decay (eq 10, solid lines in Figure 5), indicative of a diffusive process. The values of the time constant Γ obtained from the fittings follow a linear behavior with *Q*^2^, as can be observed in Figure 6. From the slope, the center-of-mass diffusion in crowded conditions was obtained using the diffusion coefficient from DLS in the dilute solution. The values of the obtained diffusion coefficient for the dilute and the crowded SCNPs are listed in Table 2. As can be observed, the diffusivity of the SCNPs is slowed down upon crowding with linear chains in a 30–35%, being slightly slower when crowding with high-*M*_w_ dPMMA.

**Table 2:** Center-of-Mass Diffusion (*D*_CM_), Effective Viscosity (η_eff_), Measured Viscosity (η), and Correlation Length (ξ) for the Systems Investigated

		*D*_CM_ (Å^2^/ns)	η_eff_ (mPa s)	η (mPa s)	ξ (Å)
SCNPs	dilute	7.17 ± 0.01^a^		0.82^b^	
	with Lo-dPMMA	2.6 ± 0.1	2.2 ± 0.2	7.5^c^	9.7 ± 0.7^d^
	with Hi-dPMMA	2.3 ± 0.1	2.4 ± 0.2	223^c^	13.0 ± 0.9^d^

a From DLS in dilute conditions.

b From capillary viscometer measurements.

c From electromagnetically spinning viscometer measurements on crowder solutions at 200 mg/mL.

d From SAXS measurements at 200 mg/mL.

We conclude that the center-of-mass diffusion is slowed-down while the internal dynamics remains unchanged. This confirms the broadening of the distribution of relaxation times observed in the analysis using a phenomenological approach (see β_w_-values in Figure 3b) and the steeper *Q*-dependence of the characteristic times with respect to the diluted case that can be observed in Figure 3a.

The values of the center-of-mass diffusion in crowded conditions can be used to make an estimation of the effective viscosity that the SCNP is sensing through the Stokes–Einstein equation
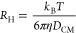
11where *R*_H_ is the hydrodynamic radius of the particle, *T* is the temperature, *k*_B_ is the Boltzmann constant, and η is the viscosity of the medium. As there is no change in the conformational parameters of the SCNP upon crowding,^12^ the *R*_H_ remains unchanged when increasing the polymer concentration, and thus, the ratio of viscosities is inversely proportional to the ratio of the diffusion coefficients of the SCNPs in the dilute solution divided by that of SCNPs in crowded conditions
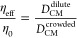
12with η_0_ being the viscosity of the neat solvent.

**Figure 7:**
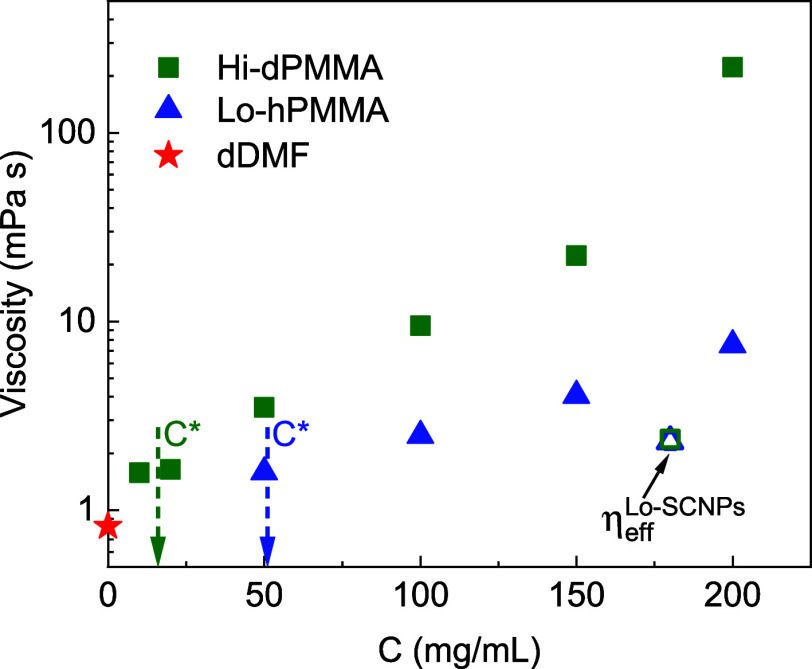
Macroscopic viscosity of solutions of PMMA crowders in DMF as a function of polymer concentration (blue triangles: Lo-hPMMA; green squares: Hi-dPMMA). Dashed arrows mark the overlap concentration for each crowder. Red star indicates the viscosity of dDMF. Open symbols indicate the effective viscosity sensed by the SCNPs under crowded conditions.

According to eq 12, the effective viscosity that the SCNPs sense when crowded with linear dPMMA is about 3 times that of dDMF, being slightly bigger in the systems crowded with long dPMMA chains (see Table 2). To compare with the macroscopic values, viscosity measurements have been carried out on solutions of PMMA in DMF at concentrations ranging between 10 and 200 mg/mL. Figure 7 displays the macroscopic viscosity of crowder solutions as a function of the polymer concentration for both low-*M*_w_ and high-*M*_w_ linear PMMA chains, along with the viscosity of neat dDMF and the effective viscosity felt by the SCNPs in crowded solutions estimated using eq 12.

At 200 mg/mL, the viscosity of the Lo-hPMMA solution is η = 7.5 mPa s, which is about 9 times that of dDMF. Moreover, in the Hi-dPMMA solutions, the viscosity is much larger, being η = 223 mPa s. Thus, the effective viscosity is clearly decoupled from the macroscopic viscosity due to the comparable size of the diffusing particles in relation to the correlation length of the crowder solution. This result can be rationalized in terms of existing theories on the diffusion of nanoparticles in polymer solutions. Initially, de Gennes et al. based on scaling theory identified three regimes according to the relative ratio between the probe particle size *R* and the polymer solution correlation length ξ.^39^ When *R*/ξ ≪ 1, the probe can simply move through the mesh and only sense the neat solvent viscosity, η_0_. On the other hand, when *R*/ξ ≫ 1, the diffusion is governed by the macroscopic viscosity, η. In the intermediate range, the local viscosity depends on the length scales probed. Experimentally, the translational diffusion has been empirically found to be a function of *R*/ξ, namely, a scaling relation *D*_0_/*D* = η_eff_/η_0_ ∼ *f*(*R*/ξ), with η_eff_ being the effective (local) viscosity, has been suggested. This has been supported by many experimental results in a wide range of NP sizes and crowder molecular weights and concentrations.^40^ In this regime, when the size of the matrix polymer is high enough, i.e., larger than the diffusing particle, diffusion is expected to be independent of the crowder molecular weight.

**Figure 8:**
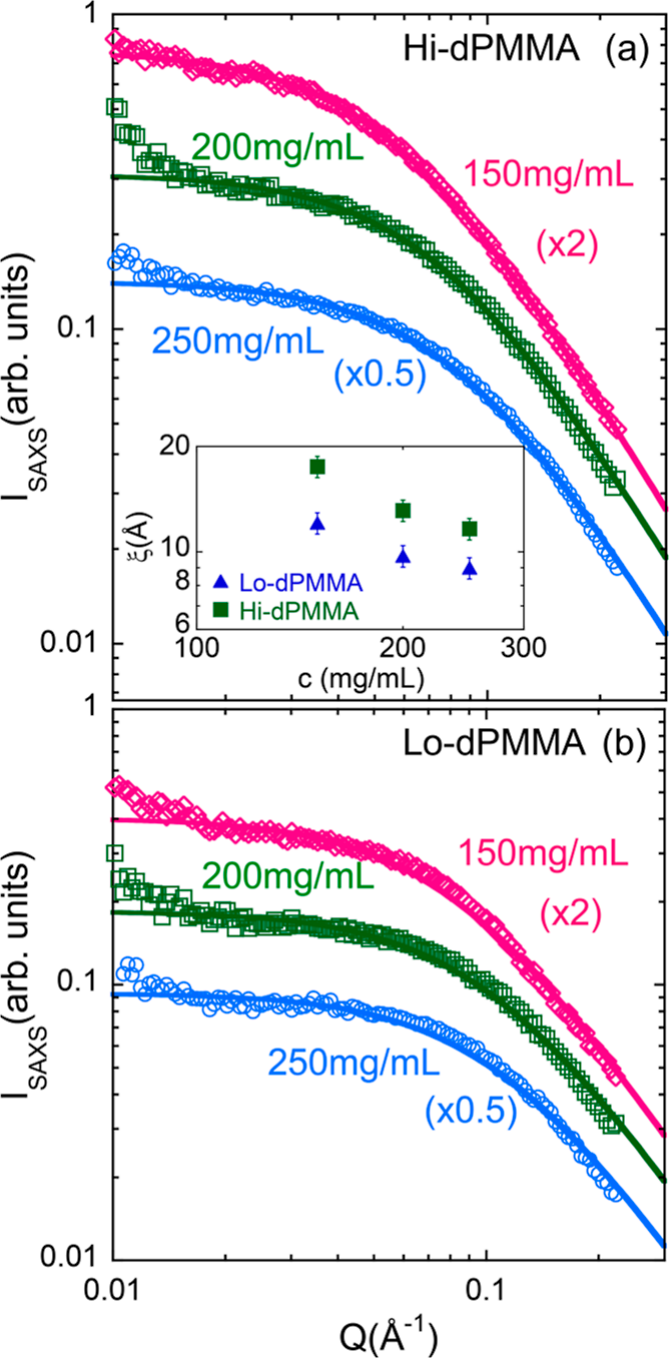
SAXS intensity as a function of Q in full contrast conditions measured for (a) Hi-dPMMA crowders and (b) Lo-dPMMA crowders in DMF solutions at the indicated concentrations (see legend). The correlation length ξ of dPMMA crowders was determined using an Orstein–Zernike function and is represented as a function of polymer concentration (blue triangles: Lo-dPMMA; green squares: Hi-dPMMA) in the inset of panel (a).

In terms of relative size, the SCNP is slightly bigger than the Lo-dPMMA but smaller than the Hi-dPMMA (see Table 1), but it is best to compare it with the crowder solution correlation length (mesh size) in our system. For that, we performed SAXS measurements and estimated the correlation length. Figure 8 shows the SAXS scattering intensities of the deuterated crowders in DMF. In SAXS, in full contrast conditions, the correlation length ξ is determined from the scattering intensity using the Ornstein–Zernike formula, *I*(*Q*) = *I*(0)/(1 + *Q*^2^ξ^2^). Small deviations at low-*Q* may be attributed to a small degree of heterogeneities (including the possibility of nanobubbles), which are absent in the SANS curves obtained in the same contrast conditions as the present NSE experiments.^12^ We note that, moreover, this feature is observed at *Q*-values out of the NSE experimental window accessed in this dynamical study. The correlation lengths as a function of crowder concentration are also depicted in the inset of Figure 8a, and the values at the crowder concentration here considered (200 mg/mL) are collected in Table 2. Both crowder solutions have a comparable correlation length, which makes the effective viscosity in both solutions very similar.

Moreover, the SCNP probes are not rigid NPs but rather soft and flexible polymer NPs. Previous work considering the fractal dimension of the diffusing particle found that globular proteins can be treated as rigid particles, whereas the diffusion of branched polymers in polymer solutions was shown to be less hindered than that of rigid spheres in the same conditions.^41^

On the other hand, the scaling theory by Cai et al.^42^ considers the effect of the crowder chain relaxation on the NP motion. At short timescales, the probe particle only feels the solvent viscosity η_0_ but from the timescale corresponding to the correlation blob relaxation time, the particle enters a subdiffusive regime due to coupling with the crowder dynamics. In this regime, the effective viscosity “felt” by the particle is time-dependent. This subdiffusive regime continues until the relaxation time of a polymer section of size comparable to that of the probe particle. Then, the effective viscosity is predicted to scale as η_eff_/η_0_ ∼ (*R*/ξ)^2^. Generally, the time dependence of the effective viscosity is not explored because the short timescales involved are normally inaccessible by DLS, FCS, or FRAP. Here, NSE probes the length and timescales relevant to the crowder dynamics. However, in our results, after factoring in the internal dynamics, there is only a diffusive process, and we do not observe such a coupling of the SCNP motion with the crowder dynamics.

## Conclusions

In summary, we studied the dynamics of SCNPs in solutions of analogous linear polymers as model systems for IDPs under crowding. We investigated SCNPs of 33 kDa—the molecular weight of an intermediate-size protein—in solutions of low- and high-molecular weight crowders. We used NSE to access the internal dynamics of the SCNPs as well as the particle diffusion.

The NSE results show that the intermediate scattering decay functions slow down for the SCNPs in crowded solutions, owing primarily to a reduction of the translational diffusion. However, the *Q*-dependence of the apparent diffusivities indicates that there is still a contribution of the SCNP chain internal modes to the overall dynamics, which persists under crowding. In dilute conditions, the dynamics of the SCNPs is well described by the Zimm model with a center-of-mass diffusion contribution superimposed to the internal dynamics, where the higher modes are suppressed due to intramolecular cross-linking, thus leaving only the rotational mode of the SCNP. The ratio of the NSE data in dilute conditions over the data corresponding to crowding conditions shows a monoexponential decay with relaxation rates that scale as *Q*^2^, suggesting a pure diffusive process. This indicates that the internal dynamics of the SCNP is essentially unaffected by the presence of the crowder. Moreover, it allows the quantification of the center-of-mass diffusion, which decreases in crowded solutions with no big effect of the crowder molecular weight. Additionally, the effective viscosity felt by the SCNP is higher than the solvent viscosity but lower than the macroscopic viscosity, in agreement with theoretical predictions and experimental observations. We do not observe any sign of coupling between the SCNP dynamics and the crowder chain dynamics at the time and length scales relevant to the crowder segmental dynamics. The nanosecond timescale in biomacromolecule solutions is important for understanding the dynamics and motions of chains at a molecular level, including conformational changes, folding, or molecular recognition.

## Supporting Information Available

Analysis of the NSE results in dilute solution in terms of the ZIF (PDF)
