# Supplementary material for: Dynamics of Single-Chain Nanoparticles under Crowding: A Neutron Spin Echo Study
Source: Macromolecules. 2024 May 7;57(10):4706–16. doi: 10.1021/acs.macromol.4c00182 (PMC11141241; doi:10.1021/acs.macromol.4c00182)
Supplement: Supplementary file 1 — ma4c00182_si_001.pdf [file ma4c00182_si_001.pdf]

Supporting Information: Dynamics of Single-Chain Nanoparticles under Crowding: a Neutron Spin Echo Study

Beatriz Robles-Hernández,[*,†] Paula Malo de Molina,[*,‡,¶] Isabel Asenjo-Sanz,[‡] Marina Gonzalez-Burgos,[‡] Stefano Pasini,[§] José A. Pomposo,[‡,¶,∥] Arantxa Arbe,[‡] and Juan Colmenero[‡,∥,†]

†*Donostia International Physics Center (DIPC), 20018 Donostia-San Sebastián, Spain*

‡*Centro de Física de Materiales/Materials Physics Center (CFM/MPC), 20018 Donostia-San Sebastián, Spain*

¶*IKERBASQUE – Basque Foundation for Science, 48009 Bilbao, Spain*

§*Forschungszentrum Jülich GmbH, Jülich Centre for Neutron Science (JCNS) at Heinz Maier-Leibnitz Zentrum (MLZ), 85748 Garching, Germany*

∥*Department of Polymers and Advanced Materials: Physics, Chemistry and Technology, University of the Basque Country UPV/EHU, 20018 Donostia-San Sebastián, Spain*

E-mail: beatriz.robles@ehu.eus; p.malodemolina@ehu.eus

S.1 Analysis of NSE Data of SCNPs in Dilute Solution in Terms of the Zimm Model with Internal Friction

The internal friction originated from diverse sources, for example, internal barriers, side-chain interactions, hindered dihedral rotations, or even hydrogen bonding, are considered in the and Zimm model with internal friction (ZIF).[S1] This ingredient is represented by a relaxation time $\tau_{\rm i}$, which is added to the time of each mode in eq 6. Thus, the resulting characteristic time for the pth mode becomes $\tau_p^{\rm ZIF} = \tau_p^{\rm Z} + \tau_{\rm i}$. The dilute solution data were analyzed in terms of this approach, using eqs 8 and 9 with $\tau_p = \tau_p^{\rm ZIF}$. When the ZIF model is applied to the dilute solution, a good description of the data is achieved with $\tau_{\rm i} = 55$ ns, in very good agreement with previous results.[S2,S3] Certainly, both modified Zimm models provide a good and nearly indistinguishable description of the data (see Figure S1).

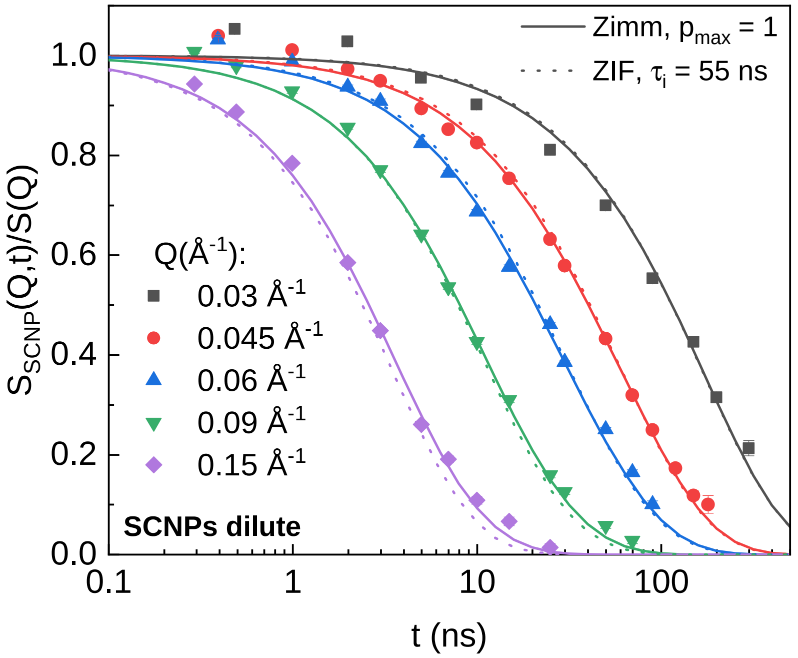

Figure S1: NSE results for SCNPs in dilute solution. Solid lines are fittings considering a Zimm model with limited mode contributions ($p_{\rm max} = 1$). The dotted lines are fittings to the ZIF model ($\tau_{\rm i} = 55$ ns).

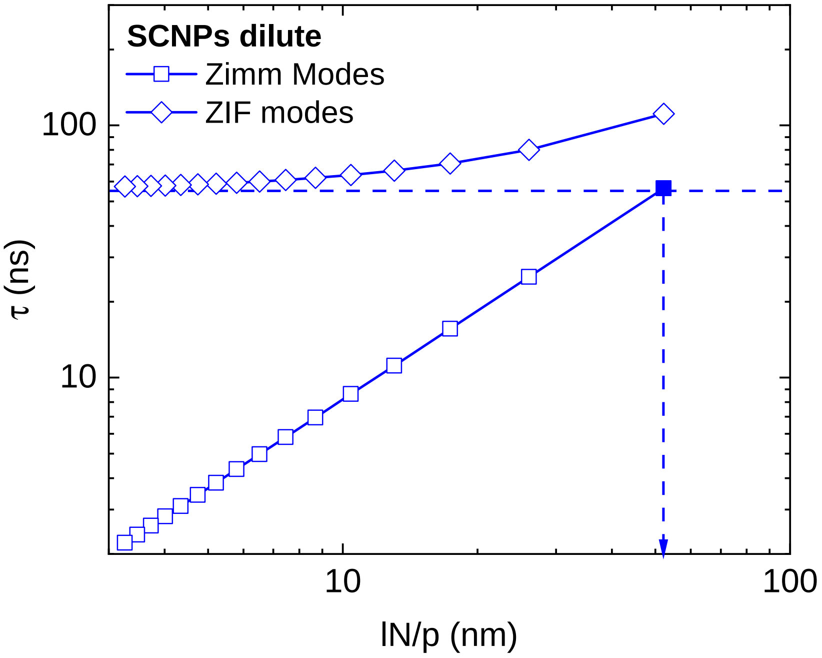

Figure S2: Dependence of the characteristic times of the Zimm modes with the wavelength for the effective chain mapping the SCNPs (blue squares) in dilute solution. Only modes with $p \leq 1$, highlighted as a filled symbol, would substantially contribute. The characteristic times for the ZIF model are represented by blue diamonds. Dashed arrows indicate the value of τ_i (55 ns) and the point where the crossover from solvent- to internal friction-dominated relaxation takes place.

The corresponding spectrum of relaxation times is represented in Figure S2. The abrupt effect produced by the mode cutoff, which can be seen as a freezing of the modes with $p > p_\mathrm{max}$ (i.e., $\tau^\mathrm{Z}_{p>p_\mathrm{max}} \equiv \infty$) is introduced more gently in the ZIF model: it is represented by a transition from solvent friction-dominated relaxation (low-p values, equivalently long-wavelength modes) to internal friction-dominated relaxation (high-p values, equivalently short-wavelength modes). This crossover occurs when $\tau^\mathrm{Z}_p \approx \tau_\mathrm{i}$ (see Figure S2).

References

(S1) Khatri, B. S.; McLeish, T. C. B. Rouse Model with Internal Friction: A Coarse Grained Framework for Single Biopolymer Dynamics. *Macromolecules* **2007**, *40*, 6770–6777.

(S2) Arbe, A.; Pomposo, J.; Moreno, A.; LoVerso, F.; González-Burgos, M.; Asenjo-Sanz, I.; Iturrospe, A.; Radulescu, A.; Ivanova, O.; Colmenero, J. Structure and dynamics of single-chain nano-particles in solution. *Polymer* **2016**, *105*, 532–544.

(S3) González-Burgos, M.; Asenjo-Sanz, I.; Pomposo, J. A.; Radulescu, A.; Ivanova, O.; Pasini, S.; Arbe, A.; Colmenero, J. Structure and Dynamics of Irreversible Single-Chain Nanoparticles in Dilute Solution. A Neutron Scattering Investigation. *Macromolecules* **2020**, *53*, 8068–8082.
